# Breast cancer diagnosis is associated with relative left ventricular hypertrophy and elevated endothelin-1 signaling

**DOI:** 10.1186/s12885-020-07217-1

**Published:** 2020-08-12

**Authors:** Zaid H. Maayah, Shingo Takahara, Abrar S. Alam, Mourad Ferdaoussi, Gopinath Sutendra, Ayman O. S. El-Kadi, John R. Mackey, Edith Pituskin, D. Ian Paterson, Jason R. B. Dyck

**Affiliations:** 1 Cardiovascular Research Centre, Department of Pediatrics, Faculty of Medicine and Dentistry, 458 Heritage Medical Research Centre, University of Alberta, Edmonton, Alberta T6G 2S2 Canada; 2 Division of Cardiovascular Surgery, Tohoku University Graduate School of Medicine, Sendai, Miyagi Japan; 3 Department of Medicine, University of Alberta, Edmonton, Alberta Canada; 4 Faculty of Pharmacy and Pharmaceutical Sciences, University of Alberta, Edmonton, Alberta Canada; 5 Cross Cancer Institute, Edmonton, Alberta Canada; 6 Division of Cardiology, Mazankowski Alberta Heart Institute, University of Alberta, Edmonton, Canada

**Keywords:** Breast cancer, Endothelin-1, Cardiac hypertrophy

## Abstract

**Background:**

The survival rates of women with breast cancer have improved significantly over the last four decades due to advances in breast cancer early diagnosis and therapy. However, breast cancer survivors have an increased risk of cardiovascular complications following chemotherapy. While this increased risk of later occurring structural cardiac remodeling and/or dysfunction has largely been attributed to the cardiotoxic effects of breast cancer therapies, the effect of the breast tumor itself on the heart prior to cancer treatment has been largely overlooked. Thus, the objectives of this study were to assess the cardiac phenotype in breast cancer patients prior to cancer chemotherapy and to determine the effects of human breast cancer cells on cardiomyocytes.

**Methods:**

We investigated left ventricular (LV) function and structure using cardiac magnetic resonance imaging in women with breast cancer prior to systemic therapy and a control cohort of women with comparable baseline factors. In addition, we explored how breast cancer cells communicate with the cardiomyocytes using cultured human cardiac and breast cancer cells.

**Results:**

Our results indicate that even prior to full cancer treatment, breast cancer patients already exhibit relative LV hypertrophy (LVH). We further demonstrate that breast cancer cells likely contribute to cardiomyocyte hypertrophy through the secretion of soluble factors and that at least one of these factors is endothelin-1.

**Conclusion:**

Overall, the findings of this study suggest that breast cancer cells play a greater role in inducing structural cardiac remodeling than previously appreciated and that tumor-derived endothelin-1 may play a pivotal role in this process.

## Background

Breast cancer survival rates have improved over the last four decades largely as the result of advances in screening leading to earlier diagnosis and improved anti-cancer therapy [1, 2]. Since cytotoxic chemotherapies continue to be central in the treatment of breast cancer and patients generally live longer after the diagnosis and treatment of their cancer, the short- and long-term effects of these therapies on the rest of the body are becoming a focus of research [3–5]. A well-characterized and potentially lethal side effect of many breast cancer chemotherapies is cardiotoxicity [3–5]. As such, considerable research effort has focussed on reducing this cardiotoxicity as these treatments are key to many curative intent regimens [3–6]. Based on the well-established cardiotoxic effects of many chemotherapies used in breast cancer treatment, the prevailing theory is that the drugs trigger a dose-dependent left ventricular (LV) remodeling that can, in the worst cases, progress to heart failure and death [3–5]. Given that monitoring of cardiac structure/function in women diagnosed with breast cancer is often not initiated until after the initiation of chemotherapy, it is currently unknown if the breast tumor itself also contributes to cardiac remodeling independently from the chemotherapy.

Breast tumor cells produce a number of soluble factors that regulate the crosstalk between cancer cell subpopulations within the breast tumor [7]. Most of these factors are not only crucial for the proliferation and progression of the tumor but also for the metastasis of breast cancer cells [8]. Among these secreted factors, endothelin-1 (ET-1) has garnered attention as a vital factor for the growth, progression and metastasis of breast cancer cells [9]. ET-1 (a 21-amino acid peptide) is produced by breast cancer cells via proteolytic cleavage of a large biological precursor molecule, big ET-1 (a 38-amino acid peptide), which is mediated by endothelin converting enzyme (ECE) [10–12]. In addition to the effect of ET-1 on cancer progression [9], ET-1 is known to contribute to other cardiovascular complications in non-cancer conditions including hypertension, left ventricular hypertrophy (LVH) and heart failure [13–16]. In agreement with this, the circulating levels of ET-1 are increased in patients with cardiac diseases as well as in animal models of cardiac hypertrophy [17–19]. In addition, inhibition of ET-1 signaling pathway reduces LVH and improves heart function in several models of heart failure suggesting a crucial role of ET-1 signaling in cardiac remodeling [19–21]. Thus, regardless of the role that ET-1 plays in breast tumor progression, there is potential that elevating breast cancer-derived ET-1 levels may contribute to breast tumor-induced LVH.

Based of the above information, the purpose of this study is to characterize LV structure and function in patients with breast cancer prior to receipt of cancer therapy. In addition, we aimed to investigate how breast cancer tumors communicate with the cardiomyocyte to induce cardiac remodeling and examine if ET-1 signaling could be the mechanism to explain LVH in breast cancer patients.

## Methods

### Materials

Human cell lines were purchased from American Type Cell Culture ((ATCC), Manassas, VA). Primary antibodies were purchased from Cell Signaling Technology (Danvers, MA), or Thermo Fisher Scientific (Waltham, MA), and secondary antibodies were from Santa Cruz Biotechnology (Santa Cruz, CA). Immunoblots were developed using Western Lightning Plus-ECL enhanced chemiluminescence substrate (Perkin Elmer, Waltham, MA). ET-1 and big ET-1 assay kits were purchased from R&D system (Minneapolis, MN).

### Retrospective cohort analysis

This retrospective investigation involved 28 female patients with newly diagnosed breast cancer recruited from a tertiary care cancer hospital approximately 45 days after surgery and prior to any systemic therapy for cancer (Cross Cancer Institute, Edmonton, Alberta, Canada). A detailed medical history was recorded at the time of recruitment and those with hypertension, diabetes, or prior cancer treatments were excluded. All participants underwent anthropometric measurements (height, weight). Body mass index (BMI) and body surface area (BSA) were calculated for each patient. Blood pressure values were obtained on the same day that the non-contrast cardiac magnetic resonance imaging (MRI) scan examination was performed. A control cohort of women with comparable baseline factors including age, BMI, BSA and health history (*n* = 17) were included.

### MRI protocol

All participants underwent a non-contrast cardiac magnetic resonance imaging (MRI) scan on a 1.5 T magnet (Siemens Healthcare, Erlangen, Germany). Image acquisition included steady-state free precession cines for cardiac structure and function and image analysis was performed by a single user using commercial software (Syngo, Siemens Healthcare). Left ventricular (LV) volumes and mass were calculated from a short axis stack of cines using a method of disks approach and were indexed to body surface area. The papillary muscles were excluded from LV mass and included in LV volume measures.

### Blood sample collection

All participants also had venipunctures at the time of their scan and their blood samples were stored in a 4 °C fridge. Following approval from our institutional review board, individual samples were accessed and separated by centrifugation at 3000 rpm for 10 min at room temperature and the plasma was frozen at − 80 °C.

### Cell culture and treatment

Human breast cancer MCF7 cells (ATCC HTB-22), T47-D (ATCC HTB-133), ZR75–1 (ATCC CRL-1500), MDA-MB-231 (ATCC HTB-2) and human left ventricular cardiomyocyte RL-14 cells (ATCC PTA-1499) were used in this study. Cells were grown in 75 cm_2_ tissue culture flasks at 37 °C under a 5% CO_2_ humidified environment. Human left ventricular cardiomyocyte RL-14 cells were maintained in DMEM/F-12, with phenol red supplemented with 12.5% fetal bovine serum, 20 M l-glutamine, 100 IU/ml penicillin G and 100 μg/ml streptomycin [22–24]. The breast cancer cell lines, MCF7 and T47-D are hormone receptor positive breast cancer phenotype [25, 26], whereas ZR75–1 is a hormone receptor positive and human epidermal growth factor receptor 2 (HER2) positive cell line [26]. In contrast, MDA-MB-231 is a triple negative breast cancer cells [25].

At 80% confluence, 8 ml of serum-free media (DMEM/F-12) was conditioned with breast cancer cells in T-75 flask (cell number count was ~ 6 × 10^6^) for 48 h, collected and centrifuged (500 xg, 5 min) [27]. Cardiomyocytes were washed twice with phosphate-buffered saline (PBS), and then incubated with 2 mL of breast cancer conditioned medium for 24 h with or without 1 μg/ml ET-1 monoclonal primary antibody (MA1–10818, Thermo Fisher Scientific) or 0.25 μM ET-1 receptor blocker, BQ-123 (ab141005, Abcam). Synthetic ET-1 (250 pM) (E7764, Sigma Aldrich) was used as a positive control. Control cardiomyocytes were treated with identical non-conditioned medium used for culturing the breast cancer cells. Cell culture-based experiments were independently replicated 4–6 times.

### Preparation of fractionated conditioned medium

Millipore Amicon Ultracel-30 K 15 ml tubes with 30 kDa filters (Fisher Scientific, Ottawa, Ontario, Canada) was used to fractionate the MCF7 breast cancer conditioned medium into 2 fractions, the lower filtrate containing proteins of less than 30 kDa and the upper concentrate containing proteins greater than 30 kDa as described previously [28].

### RNA extraction, cDNA synthesis and quantification of mRNA expression by quantitative real-time polymerase chain reaction (real time-PCR)

Total RNA was extracted using TRIzol reagent (Invitrogen®, Carlsbad, CA, USA) as described previously [29, 30]. Quantification of gene expression was performed by real time-PCR LightCycler® 480 white 384-well reaction plates in the LightCycler® 480 System (Roche Life Science), as described previously [29, 30].

### Cell surface area measurement

In order to measure cell surface area, cardiomyocytes were fixed with 4% paraformaldehyde, and stained with wheat-germ-agglutinin (WGA) conjugated with Alexa fluor 488, then mounted with ProLong Gold Antifade Mountant with 4′,6-diamidino-2-phenylindole (DAPI) (Thermo Fisher Scientific). Individual cell surface area was measured using ImageJ (NIH).

### Immunoblot analysis

Cardiomyocyte lysates were analyzed by sodium dodecyl sulfate-polyacrylamide gel electrophoresis (SDS–PAGE) and immunoblotting was performed as described previously [30]. Briefly, proteins from each treated group were diluted with 3x loading buffer (0.1 M Tris (hydroxymethyl) aminomethane (Tris)-HCl, pH 6.8, 4% sodium dodecyl sulfate (SDS), 1.5% bromophenol blue, 20% glycerol, 5% β-mercaptoethanol), boiled and loaded onto a 10% SDS-polyacrylamide gel. Samples were electrophoresed at 120 V for 2 h and separated proteins were transferred to Trans-Blot nitrocellulose membrane (0.45 μm) in a buffer containing 25 mM Tris–HCl, 192 mM glycine, and 20% (v/v) methanol. Nitrocellulose membranes from the protein transfer were blocked overnight at 4 °C in a solution containing 5% skim milk powder, 2% BSA and 0.5% Tween-20 in Tris-buffered saline (TBS) solution (0.15 M NaCl, 3 mM KCl, 25 mM Tris-base). After blocking, the membranes were washed 6 times for 1 h with TBS-Tween-20 before being incubated with a primary antibody (0.2 μg/ml) for 2 h at room temperature in TBS solution containing 0.05% (v/v) Tween-20 and 0.02% sodium azide. Incubation with peroxidase conjugated secondary antibody was carried out in blocking solution for 1 h at room temperature.

The bands were visualized using the enhanced chemiluminescence method according to the manufacturer’s instructions (GE Healthcare, Mississauga, ON). Chemiluminescent detection was performed on FUJI medical X-ray film (# 4741019291, FUJIFLIM Corporation, Japan) using OPTIMAX X-Ray Film Processor (# 117040–1411-1934, PROTEC Gmbh & Co. KG, Germany). The images were then scanned using HP Scanjet 4890 photo scanner. The intensity of the protein band was semi-quantified relative to the signals obtained for β-ACTIN protein, using ImageJ® image processing program (National Institutes of Health, Bethesda, MD, http://rsb.info.nih.gov/ij). Pre-stained protein ladder (Precision Plus Protein Kaleidoscope, Cat # 161–0375, BIO-RAD, CA, USA) was loaded, electrophoresed and transferred to Trans-Blot nitrocellulose membrane (0.45 μm) along with the samples as described above. As our pre-stained protein ladder does not react with the anti-body, we used autofluorescence tape (Radtape Plus, Cat # RADP200, Diversified Biotech, Inc.) to mark the molecular weight of the protein ladder bands close to our targeted protein.

### ET-1 and big ET-1 assays

ET-1 and big ET-1 concentrations in conditioned media of breast cancer cells and human plasma were determined by immunoassay (R & D Systems).

### Statistical analysis

Statistical analysis was carried out using GraphPad Prism software (version 7.01) (GraphPad Software, Inc., La Jolla, CA). The Kolmogorov–Smirnov test was used to assess the normality of distribution of each parameter. One-way analysis of variance (ANOVA) followed by Tukey-Kramer or Dunnett post hoc multiple comparison test, unpaired two-tailed *t*-test for normally distributed data or Mann-Whitney U test for non-normally distributed data were carried out to assess which treatment group(s) showed a significant difference from the control group. Multiple t-test comparisons were adjusted using Bonferroni method. The correlations between ET-1 or Big ET-1 levels and LV parameters were determined using Pearson’s correlation coefficient. A probability value obtained less than 0.05 is considered significant.

## Results

### Demographic and clinical parameters in patients with breast cancer and their healthy controls

BSA, BMI and health history for the patients with breast cancer and a control cohort of women are shown in Table 1. While breast cancer patients demonstrated an increased heart rate, systolic and diastolic blood pressure values did not differ between the observed groups (Table 1).

**Table 1:** Demographic and clinical parameters in patients with breast cancer and a control cohort of women with comparable baseline factors

	Healthy (n = 17)	Cancer (n = 28)	***P*** value
Age, years (mean ± SD)	59 ± 10	53 ± 10	0.15
Height, cm (mean ± SD)	166 ± 8	164 ± 6	0.25
Weight, kg (mean ± SD)	75 ± 17	75 ± 19	0.89
Body Surface Area, m^2^ (mean ± SD)	2.0 ± 0.2	1.8 ± 0.2	0.81
Ideal Body Surface area, m^2^ (mean ± SD)	2.0 ± 0.1	1.6 ± 0.1	0.72
Body Mass Index, kg/ m^2^ (mean ± SD)	27 ± 6	28 ± 7	0.26
Hypertension (%)	0	0	NA
Diabetes (%)	0	0	NA
Receptor Status, n (%)			
Estrogen receptor/Progesterone receptor	NA	26 (93%)	NA
Human epidermal growth factor receptor-2	NA	9 (32%)	NA
Triple negative	NA	1 (4%)	NA
Laterality of Cancer, n (%)			
Left	NA	12 (43%)	NA
Right	NA	14 (50%)	NA
Bilateral	NA	2 (7%)	NA
Pathologic Cancer Stage, n (%)			
1	NA	12 (43%)	NA
2	NA	13 (46%)	NA
3	NA	3 (11%)	NA
Heart Rate, beat per minute (mean ± SD)	69 ± 10	80 ± 13	0.006
Systolic Blood Pressure, mmHg (mean ± SD)	126 ± 15	130 ± 15	0.37
Diastolic Blood Pressure, mmHg (mean ± SD)	75 ± 8	72 ± 10	0.32

### Patients with breast cancer exhibit relative LVH prior to chemotherapy

**Fig. 1:**
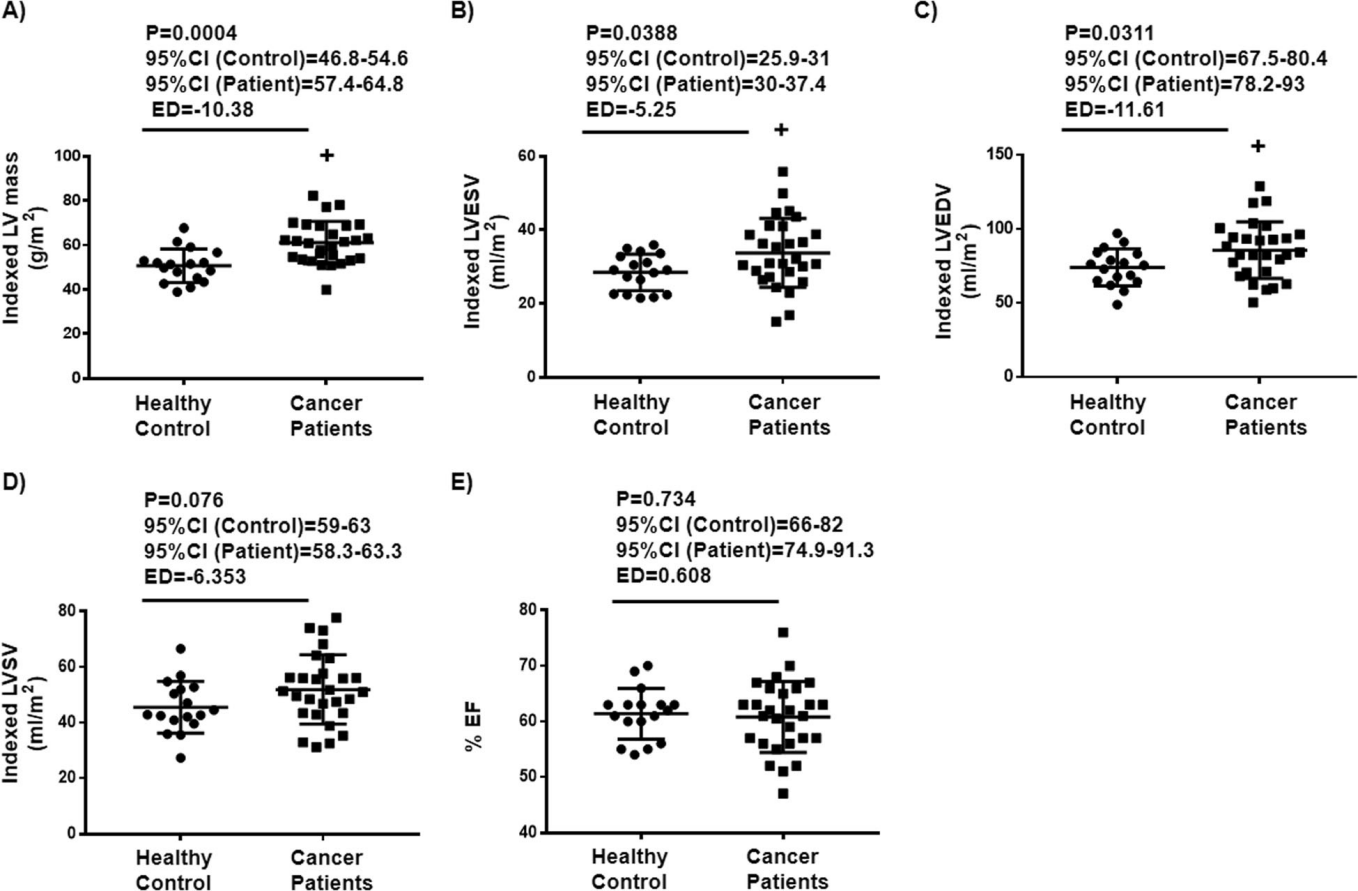
Patients with breast cancer exhibit a relative left ventricular hypertrophy prior to cancer treatment. **a** Indexed left ventricular (LV) mass. **b** Indexed left ventricular end systolic volume (LVESV), **c** Indexed left ventricular end diastolic volume (LVEDV), **d** Indexed left ventricular stroke volume (LVSV). **e** Left ventricular ejection fraction (EF) in both a control cohort of women with comparable baseline factors (*n* = 17) and breast cancer patients (*n* = 28). Dots represent individual values. Results are shown as means ± SD. Comparisons between two groups were made by unpaired *t*-test. Multiple *t*-test comparisons were adjusted using Bonferroni method. + *p* < 0.05 vs healthy control. CI: Confidence interval, ED: estimated difference

Cardiac MRI scans revealed that women with breast cancer prior to chemotherapy demonstrated signs of cardiac remodeling evidenced by the significantly increased indexed LV mass, indexed LV end-diastolic volume (LVEDV) as well as indexed LV end-systolic volume (LVESV) compared to the control cohort women (Fig. 1a, b, c). In contrast, there were no differences in LV ejection fraction (LVEF) or LV stroke volume (Fig. 1d, e) between the observed groups suggesting that patients with breast cancer can be characterized by a relative LVH with normal systolic function. While there were no differences in LVEF between breast cancer patients and the control cohort of women (Fig. 1d), 4 patients demonstrated LVEF < 55% which might occur as result of different progressive levels of LVH due to breast cancer.

### Human breast cancer conditioned medium induces hypertrophy in human cardiomyocytes

**Fig. 2:**
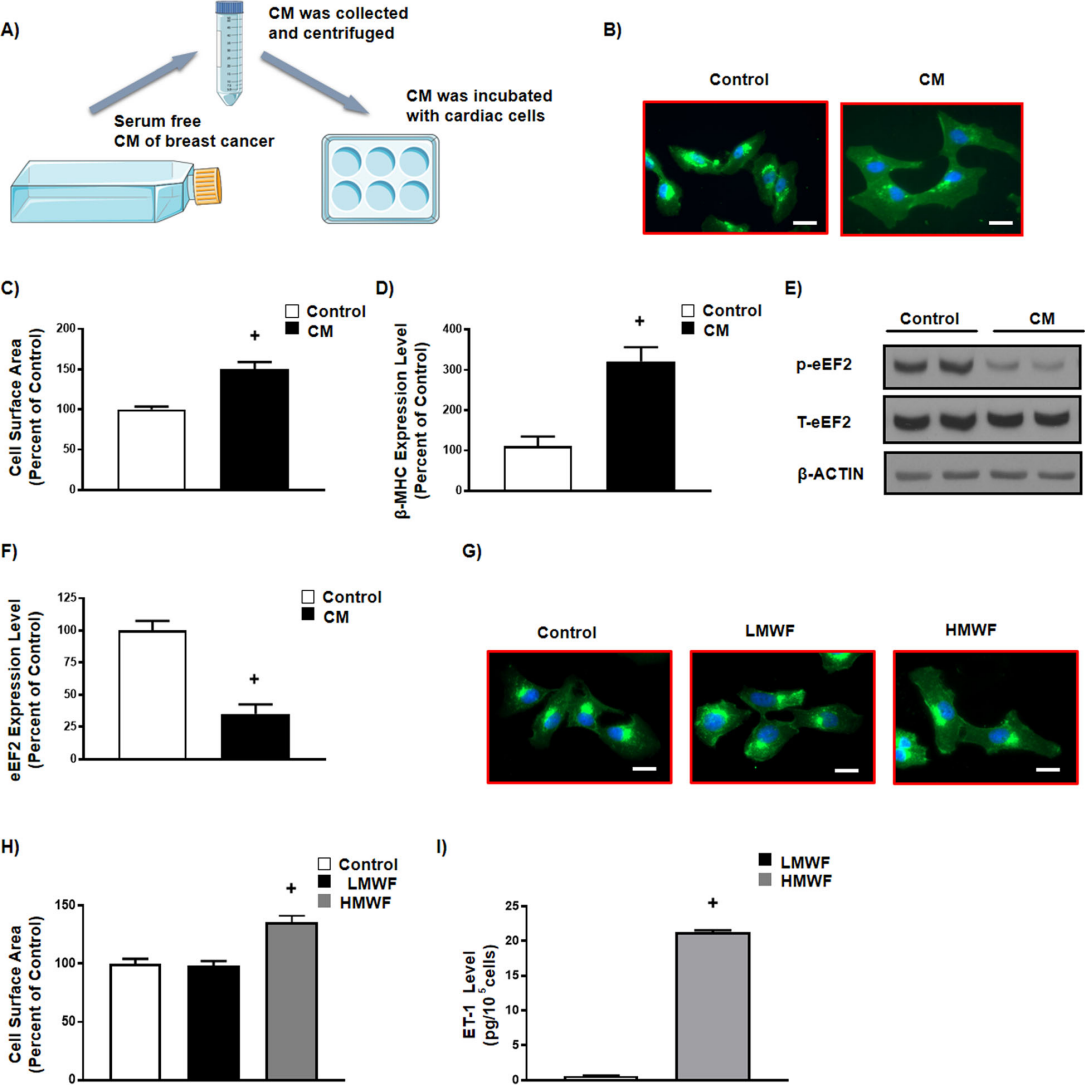
Human breast cancer conditioned medium induces hypertrophy in human left ventricular cardiomyocytes. **a** Schematic of conditioned medium (CM) treatment in human left ventricular cardiomyocytes. **b** Representative images of cardiomyocytes stained with WGA (green) and DAPI (blue), scale bar = 50 μm, **c** Quantification of cell surface area in cardiomyocytes treated with either regular serum free medium or breast cancer MCF7 CM (*n* = 70 per group). **d** β-myosin heavy chain (β-MHC) mRNA levels that were normalized to β-ACTIN in cardiomyocytes treated with either regular serum free medium or breast cancer MCF7 CM (*n* = 6). **e** Lysates from cardiomyocytes were immunoblotted with antibodies against phosphorylated eukaryotic elongation factor 2 (p-eEF2), total-eEF2 and β-ACTIN, full blot is provided in supplementary file, **f** Quantification of protein expression levels in cardiomyocytes treated with either regular serum free medium or breast cancer MCF7 CM (*n* = 4 per group). **g** Representative images of cardiomyocytes stained with WGA (green) and DAPI (blue), scale bar = 50 μm, **h** Quantification of cell surface area in cardiomyocytes treated with either regular serum free medium or low molecular weight fractionated medium (LMWF, < 30 kDa) or high molecular weight fractionated medium (HMWF, > 30 kDa) (n = 70 per group). **i** Quantification of endothelin-1 (ET-1) levels in cardiomyocytes treated with fractionated breast cancer MCF7 CM (n = 6 per group). Results are shown as means ± SEM. Comparisons between two groups were made by unpaired *t*-test, whereas comparisons between three groups were made by one-way ANOVA with a Tukey Kramer’s post hoc multiple comparison test. + *p* < 0.05 vs its own control group. Figure 2A was modified from Servier Medical Art, licensed under a Creative Common Attribution 3.0 Generic License. http://smart.servier.com/

Given that cancer was the only independent, overt clinical parameter differing between the observed groups (Table 1), we hypothesized that breast tumors cause direct detrimental alterations in cardiomyocytes that induce LVH. To test this, we incubated human LV cardiomyocytes with conditioned medium of human breast cancer MCF7 cells as described in Materials and Methods (Fig. 2a), then measured cardiomyocyte size, which is indicative of cardiomyocyte hypertrophy [31]. Of interest, we observed that conditioned medium from breast cancer MCF7 cells significantly increased cardiomyocyte size compared to cells without conditioned medium (Fig. 2b, c). The pro-hypertrophic effect of breast cancer conditioned medium was confirmed by the significantly increased pro-hypertrophic marker, β-myosin heavy chain (β-MHC) (Fig. 2d) and the significantly decreased phosphorylated eukaryotic elongation factor-2 (p-eEF2) (Fig. 2e, f and Supplementary Fig. 1A, B, C), which is indicative of elevated eEF2 activity and increased protein synthesis [31]. Together, our findings indicate that breast tumors may directly induce cardiomyocyte hypertrophy and suggest a breast cancer cell-released soluble factor may be responsible for this effect.

### Breast cancer cells secrete ET-1

To identify the breast cancer cell-released soluble factor that induces cardiomyocyte hypertrophy, we fractionated breast cancer conditioned medium utilizing Millipore Amicon Ultracel-30 K tube filtration and screened the elute fractions for inducing hypertrophy in human cardiomyocytes. Surprisingly, the hypertrophic effect was located in the fraction with molecular weight above 30 kDa (Fig. 2g, h), which is much larger than the size of humoral factors including pro-inflammatory cytokines and growth factors (Fig. 2g, h).

Since proteome analysis has already identified a number of soluble factors secreted from breast cancer conditioned medium [32–34], we used these findings to hypothesize that ET-1 is one of the candidate soluble factors that could contribute to the hypertrophic changes observed in cardiomyocytes treated with conditioned medium of human breast cancer cells. Indeed, ET-1 has direct detrimental effects on the cardiovascular system and it is known as a pro-hypertrophic factor [19, 21, 35]. Consistent with our hypothesis, the active fraction of MCF7 breast cancer conditioned medium was highly enriched with ET-1 (Fig. 2i).

### Breast cancer conditioned medium induces hypertrophy in human cardiomyocytes through the ET-1 signaling pathway

**Fig. 3:**
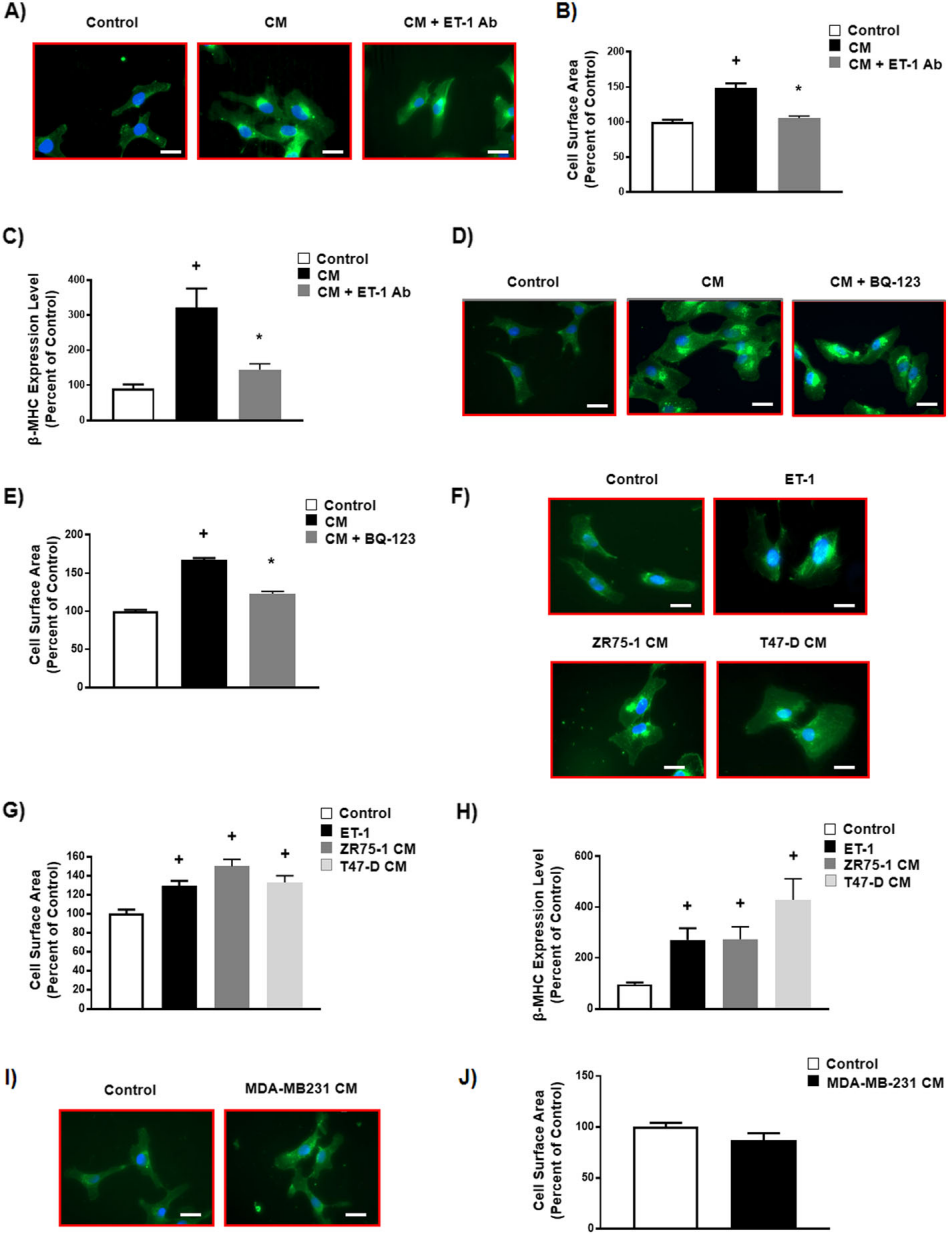
Breast cancer conditioned medium induces hypertrophy in human cardiomyocytes through endothlin-1 signaling pathway. **a** Representative images of cardiomyocytes stained with WGA (green) and DAPI (blue), scale bar = 50 μm, **b** Quantification of cell surface area in cardiomyocytes treated with either regular serum free medium or breast cancer MCF7 CM with or without the antibody against ET-1 (n = 70 per group). **c** β-MHC mRNA levels that were normalized to β-ACTIN in cardiomyocytes treated with either regular serum free medium or breast cancer MCF7 CM with or without antibodies against ET-1 (n = 6 per group), **d** Representative images of cardiomyocytes stained with WGA (green) and DAPI (blue), scale bar = 50 μm, **e** Quantification of cell surface area levels in cardiomyocytes treated with either regular serum free medium and breast cancer MCF7 CM with or without BQ-1 (n = 70 per group), **f** Cardiomyocytes were stained with WGA (green) and DAPI (blue), scale bar = 50 μm, **g** Quantification of cell surface area in cardiomyocytes treated with regular serum free medium, synthetic ET-1, breast cancer ZR75–1 CM or breast cancer T47D CM. **h** β-MHC mRNA levels that were normalized to β-ACTIN in cardiomyocytes treated with regular serum free medium, synthetic ET-1, breast cancer ZR75–1 CM or breast cancer T47D CM (n = 6 per group). **i** Representative images of cardiomyocytes stained with WGA (green) and DAPI (blue), scale bar = 50 μm, **j** Quantification of cell surface area in cardiomyocytes treated with regular serum free medium or triple negative breast cancer MDA-MB-231 CM. Results are shown as means ± SEM. Comparisons between two groups were made by unpaired *t*-test, whereas comparisons between three groups were made by one-way ANOVA with a Tukey Kramer’s post hoc multiple comparison test. + *p* < 0.05 vs its own control group. * *p* < 0.05 vs its CM

To determine whether ET-1 is responsible for the pro-hypertrophic effect of breast cancer cells, we treated human ventricular cardiomycytes with conditioned medium of breast cancer MCF7 cells with or without the inclusion of neutralizing antibody against ET-1. Interestingly, we found that the neutralizing antibody against ET-1 blocked breast cancer conditioned medium-induced increases in cardiomyocyte size and abolished the upregulation of the pro-hypertrophic marker, β-MHC (Fig. 3a, b, c). These data indicate that ET-1 is required for the pro-hypertrophic effect of breast cancer conditioned medium.

Because ET-1 induces cardiomyocyte hypertrophy through activation of ET type A receptor (ET_A_ receptor) [21], we examined whether breast cancer conditioned medium-induced cardiomyocyte hypertrophy was dependent on the activation of ET_A_ receptors. To do this, we incubated human ventricular cardiomyocytes with conditioned medium of breast cancer MCF7 cells with or without the ET_A_ receptor blocker, BQ-123. We observed that BQ-123 repressed breast cancer conditioned medium-induced increases in cardiomyocyte size (Fig. 3d, e). Overall, these findings indicate that conditioned medium of breast cancer MCF7 cells induce cardiomyocyte hypertrophy through an ET-1-dependent signaling pathway.

### Conditioned medium of other human breast cancer cells that secrete ET-1 also induce cardiomyocytes hypertrophy

We further examined whether the pro-hypertrophic effect observed in response to conditioned medium of breast cancer MCF-7 cells is also induced by conditioned medium of other human breast cancer cells that secret ET-1, such as T47-D and ZR75–1 cells (Table 2) [34]. To do this, we incubated human ventricular cardiomyocytes with conditioned medium of breast cancer T47-D and ZR75–1 cells and compared these findings to the effects observed using synthetic ET-1 as a positive control. Our results showed that synthetic ET-1 and conditioned medium of breast cancer T47-D and ZR75–1 cells significantly increased cardiomyocyte size and the pro-hypertrophic marker, β-MHC (Fig. 3f, g, h). In contrast, conditioned medium of other human breast cancer cells that do not secret ET-1, such as MDA-MB-231 cells (Table 2) [34], did not induce cardiomyocyte hypertrophy (Fig. 3i, j). Our data are consistent with previous reports showing that other triple negative breast cancer cells such as BT549 and MDA-MB-435 s had no secretion of ET-1, suggesting that triple negative breast cancer cells generally have no secretion of ET-1 [34]. Together, these data confirm that ET-1 is a soluble factor that induces the pro-hypertrophic changes of cardiomyocytes in response to conditioned medium of breast cancer cells that secrete ET-1.

**Table 2:** The level of endothelin-1 in conditioned medium of breast cancer cell lines

Cell Line	ET-1, pg/ml/10^**5**^ cells/48 h (mean ± SEM)
MCF7	21.24 ± 0.82
ZR75–1	126.03 ± 11.95
T47-D	254.62 ± 5.91
MDA-MB-231	Non-detected

### High circulating levels of ET-1 in breast cancer patients with a relative LVH

**Fig. 4:**
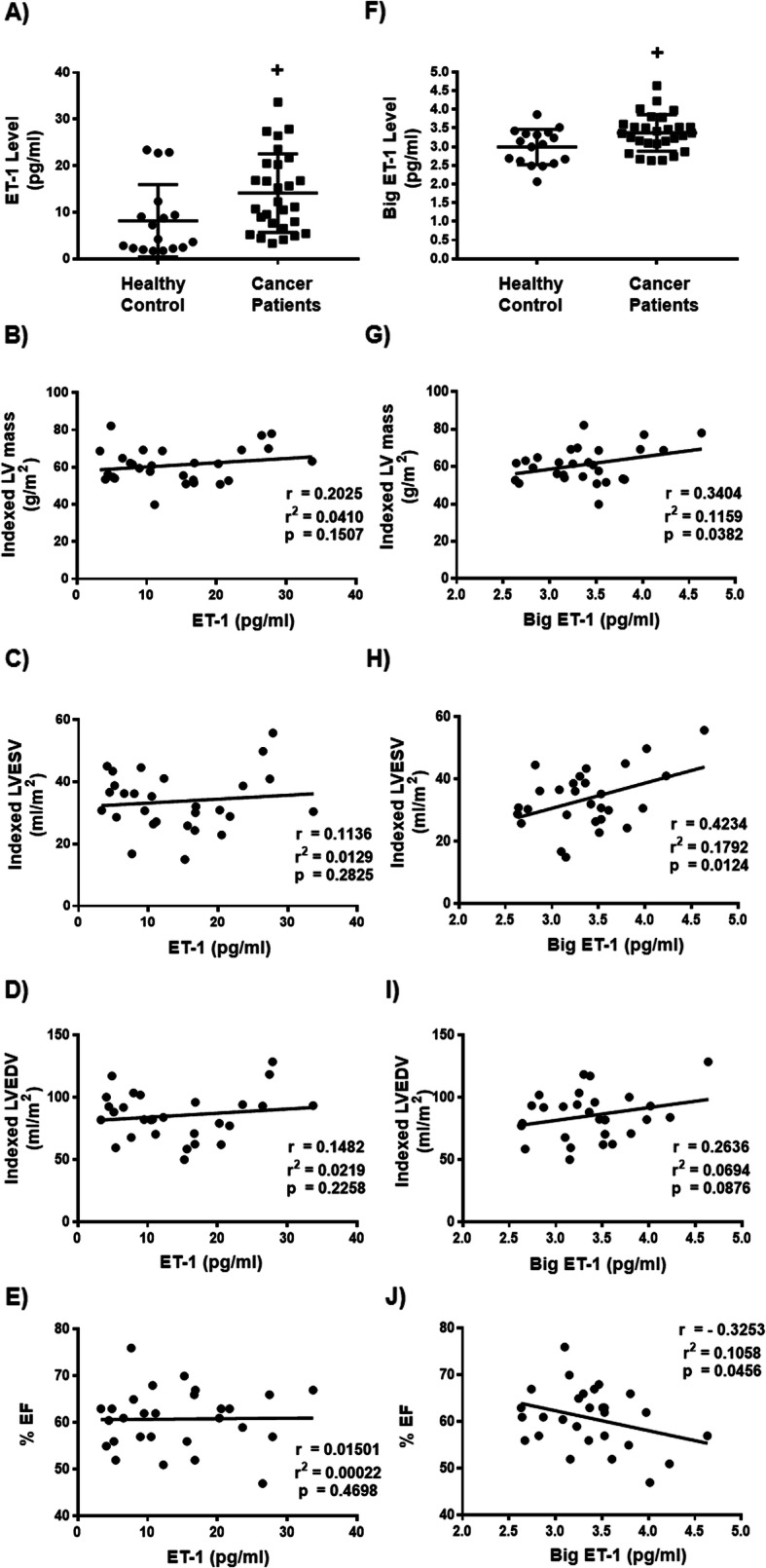
High circulating level of endothelin-1 and big endothelin-1 in patients with breast cancer with relative left ventricular hypertrophy. **a** Quantification of endothelin-1 (ET-1) levels in both breast cancer patients (*n* = 28) and a control cohort of women with comparable baseline factors (*n* = 17). **b** Correlation between ET-1 concentration and indexed left ventricular (LV) mass, **c** Correlation between ET-1 concentration and indexed left ventricular end systolic volume (LVESV), **d** Correlation between ET-1 concentration and indexed left ventricular end diastolic volume (LVEDV), **e** Correlation between ET-1 concentration and left ventricular ejection fraction (LVEF) in breast cancer patients (n = 28). **f** Quantification of big ET-1 levels in in both breast cancer patients (n = 28) and the control cohort (n = 17). **g** Correlation between big ET-1 concentration and indexed LV mass, **h** Correlation between big ET-1 concentration and indexed LVESV, **i** Correlation between big ET-1 concentration and indexed LVEDV, **j** Correlation between big ET-1 concentration and LVEF in breast cancer patients (n = 28). Dots represent individual values. Results are shown as means ± SD. Comparisons between two groups were made by unpaired *t*-test. * *p* < 0.05 vs healthy control. The correlations between ET-1 or Big ET-1 levels and LV parameters was determined using Pearson’s correlation coefficient

Since ET-1 is implicated as a crucial factor for the development of cardiomyocyte hypertrophy in response to conditioned medium of certain breast cancer cells, we measured circulating ET-1 in our cohort of breast cancer patients and the control cohort of women. Of interest, we found that breast cancer patients demonstrated higher circulating level of ET-1 compared with the control cohorts, suggesting that ET-1 may contribute to a relative LVH in breast cancer patients (Fig. 4a). However, we could not find a significant correlation between ET-1 and a relative LVH in our breast cancer patients (Fig. 4b, c, d, e), which is not surprising since ET-1 has short half-life in the systemic circulation [20].

### High circulating level of big ET-1 in breast cancer patients with a relative LVH

Given that big ET-1 is more stable than ET-1 [36] and that increased plasma levels of ET-1 are credited mainly to an increase in the level of big ET-1 [20], we measured big ET-1 in breast cancer patients with a relative LVH and controls. We found that our breast cancer patients with a relative LVH exhibited significantly higher circulating levels of big ET-1 compared with control women (Fig. 4f), demonstrating that the endothelin system is activated in the breast cancer patients with a relative LVH. Of interest, big ET-1 levels correlated positively with indexed LV mass (Fig. 4g) and LVESV (Fig. 4h) in the breast cancer patients. On the other hand, while a significant inverse relationship was obtained with LVEF (Fig. 4j), we could not find a significant correlation between big ET-1 and LVEDV (Fig. 4i). Together, these data suggest that cardiac remodeling in breast cancer patients can, in part, be attributed to the activation of endothelin system.

**Fig. 5:**
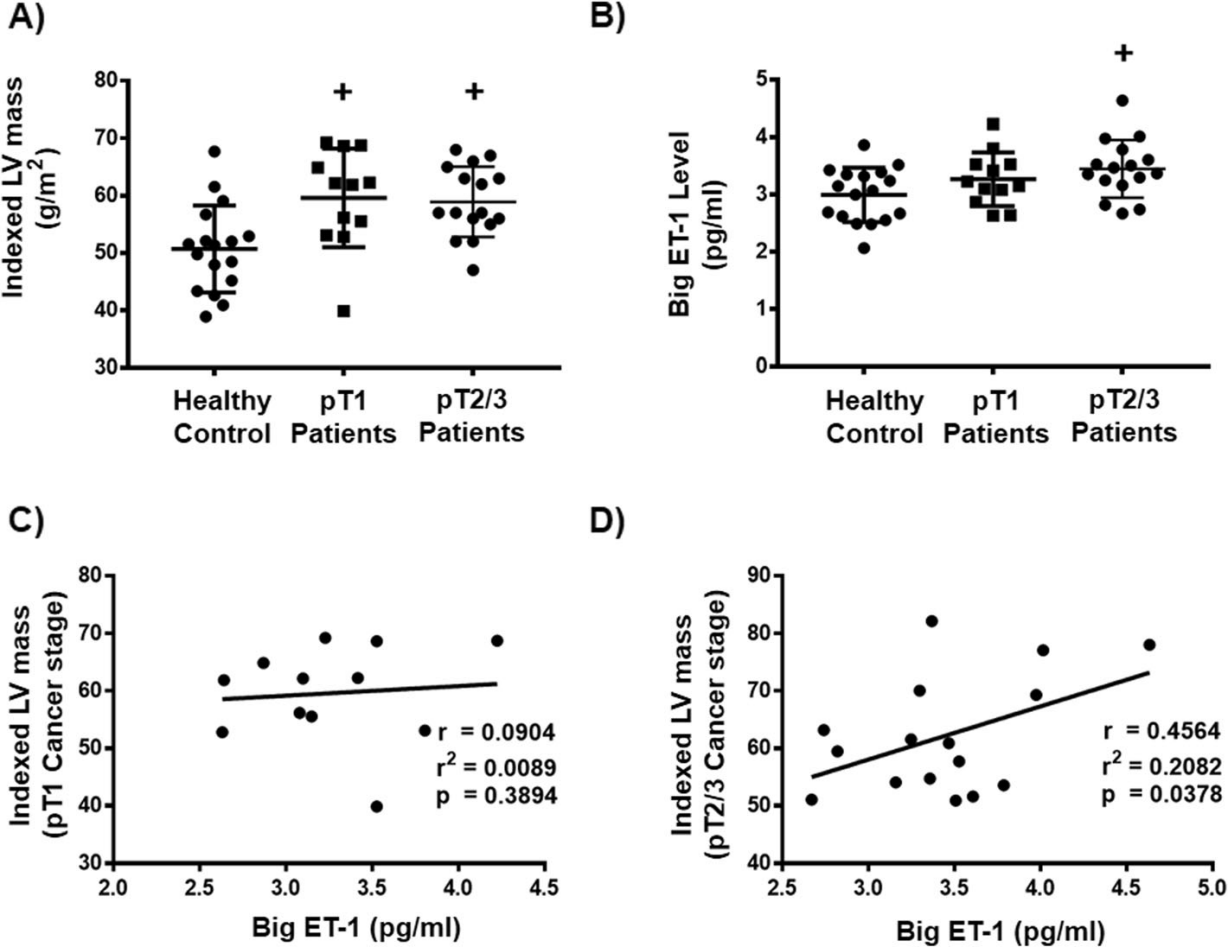
Circulating level of big endothelin-1 correlated with pT stage of breast cancer patients with relative left ventricular hypertrophy. **a** Indexed left ventricular (LV) mass in pT1 stage breast cancer patients (*n* = 12), pT2/3 stage breast cancer patients (*n* = 16) and a control cohort of women with comparable baseline factors (n = 17). **b** Big ET-1 concentration in pT1 stage breast cancer patients (n = 12), pT2/3 stage breast cancer patients (n = 16) and a control cohort of women with comparable baseline factors (n = 17). **c** Correlation between big ET-1 concentration and indexed LV mass in pT1 stage breast cancer patients (n = 12), **d** Correlation between big ET-1 concentration and indexed LV mass in pT2/3 stage breast cancer patients (n = 16). Dots represent individual values. Results are shown as means ± SD. Comparisons between three groups were made by one-way ANOVA with a Dunnett post hoc multiple comparison test. +*p* < 0.05 vs healthy control. The correlations between Big ET-1 levels and indexed LV mass in pT stage of breast cancer patients was determined using Pearson’s correlation coefficient

Notwithstanding the previous finding, while pT1 and pT2/3 stage patients demonstrated signs of cardiac remodeling evidenced by the significantly increased indexed LV mass (Fig. 5a), big ET-1 levels significantly increased and correlated positively with indexed LV mass in pT2/3 but not pT1 stage patients (Fig. 5b, c, d) suggesting that the activation of ET-1 signaling is more pronounced in advanced stages of breast cancer.

## Discussion

In an interesting and exciting new discovery, we show that even prior to systemic therapy for breast cancer, patients diagnosed with breast cancer exhibit LV remodeling characterized by relative hypertrophy. We also provide evidence suggesting that breast tumors communicate directly with cardiomyocytes to induce this phenotype. Our findings support the hypothesis that the tumor itself can have a detrimental effect on the heart [37]. This notion is important as breast cancer survivors are at high risk for cardiac disease [6, 38]. Considering the fact that risk stratification of cancer survivors is problematic [39], understanding the pathogenesis of the cardiovascular risk associated with a breast cancer diagnosis may improve the identification of susceptible patients.

Given the fact that; 1) patients diagnosed with breast cancer exhibit LV remodeling, and 2) conditioned medium of breast cancer cells induces hypertrophy in human cardiomyocytes, we postulated that breast tumor cells secrete soluble factors that cause detrimental alterations in important signaling pathways in cardiomyocytes that induce relative LVH. While breast cancer cells secrete a number of pro-inflammatory cytokines and growth factors that contribute to the progression and metastasis of cancer [32], our data show that none of these factors are implicated in the development of cardiomyocyte hypertrophy in response to breast tumor cells. Thus, these findings indicate that the observed cardiomyocyte hypertrophy is not due to inflammation and/or the secretion of small molecules such as pro-inflammatory cytokines and growth factors, suggesting that the cardiomyocyte response is due to active peptides secreted by the breast cancer cells.

ET-1 is an active peptide that has garnered much attention over the last decade as an important factor for the progression and metastasis of breast cancer [33]. Interestingly, ET-1 is also known as a pro-hypertrophic factor [21] and its circulating level is increased in patients with LVH and heart failure [19, 35]. Thus, it is likely that ET-1 could be one of the candidate soluble factors that govern the hypertrophic changes of cardiomyocytes. Consistent with this notion, only conditioned medium of human breast cancer cells that secrete ET-1 were able to induce cardiomyocyte hypertrophy. In addition, this hypertrophy was abolished by either an ET-1 antibody or an ET-1 receptor blocker indicating that the induction of cardiomyocytes hypertrophy by conditioned medium from breast tumor cells is mediated through the ET-1 signaling pathway.

In order to examine whether the effect observed in the cell culture model holds true in the clinical setting, we measured the circulating levels of ET-1 in our breast cancer patients and their controls. While the circulating levels of ET-1 was higher in our breast cancer patients compared to control women, we could not find a significant correlation between LV mass and ET-1 within the patient group. The lack of a correlation might be attributed to the fact that ET-1 is unstable as it is degraded in the circulation or is rapidly taken up by the vasculature [20]. Thus, it is likely that measured levels of circulating ET-1 from a distal appendage might underestimate the amount of ET-1 located in closer proximity to the tumor. This is in agreement with the notion that absolute ET-1 levels in the blood may be an inaccurate indicator of endothelin system activation [40]. Nevertheless, the significantly increased circulating level of ET-1 in our breast cancer patients suggests that ET-1 contributes to LV remodeling in breast cancer patients [19].

Big ET-1 is more stable than ET-1 as it has a longer half-life and it is cleared slowly from the circulation [36, 41]. Thus, big ET-1 is considered to be a sensitive measure of endothelin system activation [41]. Given that the high circulating level of ET-1 is mainly attributed to the release of big ET-1 [36, 41], we measured big ET-1 in our breast cancer patients with relative LVH and healthy control women. Interestingly, the high levels of big ET-1 in our breast cancer patients was positively correlated with LV volume and mass. These findings not only confirm that the activation of endothelin system may play a role in mediating relative LVH in breast cancer patients but also indicate that big ET-1 is a sensitive biomarker that may help clinicians identify patients at higher risk for subsequent cardiac remodelling. In agreement with our results, plasma big ET-1 is increased in patients with heart failure or cardiomyopathy and is significantly correlated with the extent of cardiac remodeling [36, 41, 42]. Thus, plasma big ET-1 is recommended as a prognostic biomarker of hypertrophic cardiomyopathy due to endothelin system activation [42].

## Conclusion

Our results indicate that even prior to cancer treatment, breast cancer patients already exhibit relative LVH. We show that breast tumor cells may contribute to cardiomyocytes hypertrophy through the secretion of soluble factors and that one of these major factors is ET-1. While we have determined the level of ET-1 and big ET-1 in breast cancer patients, this may not reflect the actual levels of these factors produced by tumors as 24 of 28 of our breast cancer patients had tumour resection approximately 45 days prior to the cardiac MRI measure. Nevertheless, our data provided insight into a potential mechanism that may involve breast cancer cell-secreted ET-1 causing cardiomyocyte cell growth. Thus, given the fact that LVH is strongly linked to increased risk of heart failure in clinical studies [42], targeting ET-1 signaling pathway and LVH may help minimize the susceptibility of patients with breast cancer to cardiovascular disease and ultimately improve their survival.

An important limitation of the current study is that we used a relatively small number of patients and healthy control women and thus, these findings should be confirmed in a larger cohort of patients and a control cohort with comparable baseline factors. Although we found that ET-1 signaling is increased in our breast cancer patients, we have not determined the level of residual tumor in these patients. Nonetheless, circulating ET-1 level might not accurately reflect the amount of ET-1 located in closer proximity to the tumor. Moreover, we have not explored the effect of other factors such as a stress-related increase in catecholamines on LVH and ET-1 signaling in our breast cancer patients. Lastly, additional studies should explore the clinical implications of relative LVH and increased ET-1 signaling in breast cancer patients during cancer treatment and beyond.

## Supplementary information

**Additional file 1 Supplementary Fig. 1.** The original, full blot for phosphorylated eukaryotic elongation factor-2 (p-eEF2), total-eEF2 and β-ACTIN.

## Data Availability

The data used or analyzed during this study are included in this article and available from the corresponding author upon reasonable request.

## Abbreviations

ATCC: American Tissue Culture Collection
BMI: Body mass index
BSA: Body surface area
CM: Conditioned medium
DAPI: 4′,6-diamidino-2-phenylindole
EF: Ejection fraction
ECE: Endothelin converting enzyme
ET-1: Endothelin-1
HER2: Human epidermal growth factor receptor 2
HMWF: High molecular weight fractionated medium
LV: Left ventricular
LVH: Left ventricular hypertrophy
LVEDV: Left ventricular end diastolic volume
LVESV: Left ventricular end systolic volume
LVSV: Left ventricular stroke volume
LMWF: Low molecular weight fractionated medium
MRI: Non-contrast cardiac magnetic resonance imaging
SDS–PAGE: Sodium dodecyl sulfate-polyacrylamide gel electrophoresis
p-eEF2: Phosphorylated eukaryotic elongation factor 2
WGA: Wheat-germ-agglutinin
β-MHC: β-myosin heavy chain

## References

[1] Soerjomataram I, Louwman MW, Ribot JG, Roukema JA, Coebergh JW (2008). An overview of prognostic factors for long-term survivors of breast cancer. Breast Cancer Res Treat.

[2] Dong G, Wang D, Liang X, Gao H, Wang L, Yu X (2014). Factors related to survival rates for breast cancer patients. Int J Clin Exp Med.

[3] Keefe DL (2002). Trastuzumab-associated cardiotoxicity. Cancer.

[4] Ky B, Vejpongsa P, Yeh ET, Force T, Moslehi JJ (2013). Emerging paradigms in cardiomyopathies associated with cancer therapies. Circ Res.

[5] Groarke J, Tong D, Khambhati J, Cheng S, Moslehi J (2012). Breast cancer therapies and cardiomyopathy. Med Clin North Am.

[6] Jones LW, Haykowsky MJ, Swartz JJ, Douglas PS, Mackey JR (2007). Early breast cancer therapy and cardiovascular injury. J Am Coll Cardiol.

[7] Rosen JM, Roarty K (2014). Paracrine signaling in mammary gland development: what can we learn about intratumoral heterogeneity?. Breast Cancer Res.

[8] Waning DL, Guise TA (2014). Molecular mechanisms of bone metastasis and associated muscle weakness. Clin Cancer Res.

[9] Sin A, Tang W, Wen CY, Chung SK, Chiu KY (2015). The emerging role of endothelin-1 in the pathogenesis of subchondral bone disturbance and osteoarthritis. Osteoarthr Cartil.

[10] Patel KV, Schrey MP (1995). Human breast cancer cells contain a phosphoramidon-sensitive metalloproteinase which can process exogenous big endothelin-1 to endothelin-1: a proposed mitogen for human breast fibroblasts. Br J Cancer.

[11] Yanagisawa M, Kurihara H, Kimura S, Tomobe Y, Kobayashi M, Mitsui Y (1988). A novel potent vasoconstrictor peptide produced by vascular endothelial cells. Nature.

[12] Levin ER (1995). Endothelins. N Engl J Med.

[13] Frank D, Kuhn C, Brors B, Hanselmann C, Ludde M, Katus HA (2008). Gene expression pattern in biomechanically stretched cardiomyocytes: evidence for a stretch-specific gene program. Hypertension.

[14] Sugden PH, Clerk A (2005). Endothelin signalling in the cardiac myocyte and its pathophysiological relevance. Curr Vasc Pharmacol.

[15] Schwebe M, Ameling S, Hammer E, Monzel JV, Bonitz K, Budde S (2015). Protective effects of endothelin receptor a and B inhibitors against doxorubicin-induced cardiomyopathy. Biochem Pharmacol.

[16] Bien S, Riad A, Ritter CA, Gratz M, Olshausen F, Westermann D (2007). The endothelin receptor blocker bosentan inhibits doxorubicin-induced cardiomyopathy. Cancer Res.

[17] Chien KR, Knowlton KU, Zhu H, Chien S (1991). Regulation of cardiac gene expression during myocardial growth and hypertrophy: molecular studies of an adaptive physiologic response. FASEB J.

[18] Cameron VA, Rademaker MT, Ellmers LJ, Espiner EA, Nicholls MG, Richards AM (2000). Atrial (ANP) and brain natriuretic peptide (BNP) expression after myocardial infarction in sheep: ANP is synthesized by fibroblasts infiltrating the infarct. Endocrinology.

[19] Valero-Munoz M, Li S, Wilson RM, Boldbaatar B, Iglarz M, Sam F. Dual endothelin-a/endothelin-B receptor blockade and cardiac remodeling in heart failure with preserved ejection fraction. Circ Heart Fail. 2016;9(11):e003381. 10.1161/CIRCHEARTFAILURE.116.003381 PMC5584628 27810862

[20] Wei CM, Lerman A, Rodeheffer RJ, McGregor CG, Brandt RR, Wright S (1994). Endothelin in human congestive heart failure. Circulation.

[21] Archer CR, Robinson EL, Drawnel FM, Roderick HL (2017). Endothelin-1 promotes hypertrophic remodelling of cardiac myocytes by activating sustained signalling and transcription downstream of endothelin type a receptors. Cell Signal.

[22] Mahrouf-Yorgov M, Augeul L, Da Silva CC, Jourdan M, Rigolet M, Manin S (2017). Mesenchymal stem cells sense mitochondria released from damaged cells as danger signals to activate their rescue properties. Cell Death Differ.

[23] Kobashigawa LC, Xu YC, Padbury JF, Tseng YT, Yano N (2014). Metformin protects cardiomyocyte from doxorubicin induced cytotoxicity through an AMP-activated protein kinase dependent signaling pathway: an in vitro study. PLoS One.

[24] Maayah ZH, Elshenawy OH, Althurwi HN, Abdelhamid G, El-Kadi AO (2015). Human fetal ventricular cardiomyocyte, RL-14 cell line, is a promising model to study drug metabolizing enzymes and their associated arachidonic acid metabolites. J Pharmacol Toxicol Methods.

[25] Mota AL, Evangelista AF, Macedo T, Oliveira R, Scapulatempo-Neto C, Vieira RA (2017). Molecular characterization of breast cancer cell lines by clinical immunohistochemical markers. Oncol Lett.

[26] Conley SJ, Bosco EE, Tice DA, Hollingsworth RE, Herbst R, Xiao Z (2016). HER2 drives mucin-like 1 to control proliferation in breast cancer cells. Oncogene.

[27] Zhang G, Liu Z, Ding H, Zhou Y, Doan HA, Sin KWT (2017). Tumor induces muscle wasting in mice through releasing extracellular Hsp70 and Hsp90. Nat Commun.

[28] Bairwa SC, Rajapurohitam V, Gan XT, Mangat R, Proctor SD, Karmazyn M (2016). Cardiomyocyte Antihypertrophic effect of adipose tissue conditioned medium from rats and its abrogation by obesity is mediated by the leptin to adiponectin ratio. PLoS One.

[29] Maayah ZH, Levasseur J, Siva Piragasam R, Abdelhamid G, Dyck JRB, Fahlman RP (2018). 2-Methoxyestradiol protects against pressure overload-induced left ventricular hypertrophy. Sci Rep.

[30] Maayah ZH, Althurwi HN, Abdelhamid G, Lesyk G, Jurasz P, El-Kadi AO (2016). CYP1B1 inhibition attenuates doxorubicin-induced cardiotoxicity through a mid-chain HETEs-dependent mechanism. Pharmacol Res.

[31] Matsumura N, Robertson IM, Hamza SM, Soltys CM, Sung MM, Masson G (2017). A novel complex I inhibitor protects against hypertension-induced left ventricular hypertrophy. Am J Physiol Heart Circ Physiol.

[32] Kulasingam V, Diamandis EP (2007). Proteomics analysis of conditioned media from three breast cancer cell lines: a mine for biomarkers and therapeutic targets. Mol Cellular Proteomics.

[33] Ratna A, Das SK. Endothelin: ominous player in breast cancer. J Cancer Clin Trials. 2016;1(1):e102. PMC5460525 28597003

[34] Yin JJ, Mohammad KS, Kakonen SM, Harris S, Wu-Wong JR, Wessale JL (2003). A causal role for endothelin-1 in the pathogenesis of osteoblastic bone metastases. Proc Natl Acad Sci U S A.

[35] Kinugawa T, Kato M, Ogino K, Osaki S, Igawa O, Hisatome I (2003). Plasma endothelin-1 levels and clinical correlates in patients with chronic heart failure. J Card Fail.

[36] Pacher R, Bergler-Klein J, Globits S, Teufelsbauer H, Schuller M, Krauter A (1993). Plasma big endothelin-1 concentrations in congestive heart failure patients with or without systemic hypertension. Am J Cardiol.

[37] Tadic M, Genger M, Baudisch A, Kelle S, Cuspidi C, Belyavskiy E (2018). Left ventricular strain in chemotherapy-naive and radiotherapy-naive patients with cancer. Can J Cardiol.

[38] Bradshaw PT, Stevens J, Khankari N, Teitelbaum SL, Neugut AI, Gammon MD (2016). Cardiovascular disease mortality among breast cancer survivors. Epidemiology.

[39] Watson EK, Rose PW, Neal RD, Hulbert-Williams N, Donnelly P, Hubbard G (2012). Personalised cancer follow-up: risk stratification, needs assessment or both?. Br J Cancer.

[40] Chen J, Chen MH, Guo YL, Zhu CG, Xu RX, Dong Q (2015). Plasma big endothelin-1 level and the severity of new-onset stable coronary artery disease. J Atheroscler Thromb.

[41] Rivera M, Cortes R, Portoles M, Valero R, Sancho-Tello MJ, Martinez-Dolz L (2005). Plasma concentration of big endothelin-1 and its relation with plasma NT-proBNP and ventricular function in heart failure patients. Rev Esp Cardiol.

[42] Wang Y, Tang Y, Zou Y, Wang D, Zhu L, Tian T (2017). Plasma level of big endothelin-1 predicts the prognosis in patients with hypertrophic cardiomyopathy. Int J Cardiol.

