# Supplementary material for: Breast cancer diagnosis is associated with relative left ventricular hypertrophy and elevated endothelin-1 signaling
Source: BMC Cancer. 2020 Aug 12;20:751. doi: 10.1186/s12885-020-07217-1 (PMC7425133; doi:10.1186/s12885-020-07217-1)
Supplement: Supplementary file 1 — Additional file 1 Supplementary Fig. 1. The original, full blot for phosphorylated eukaryotic elongation factor-2 (p-eEF2), total-eEF2 and β-ACTIN. [file 12885_2020_7217_MOESM1_ESM.docx]

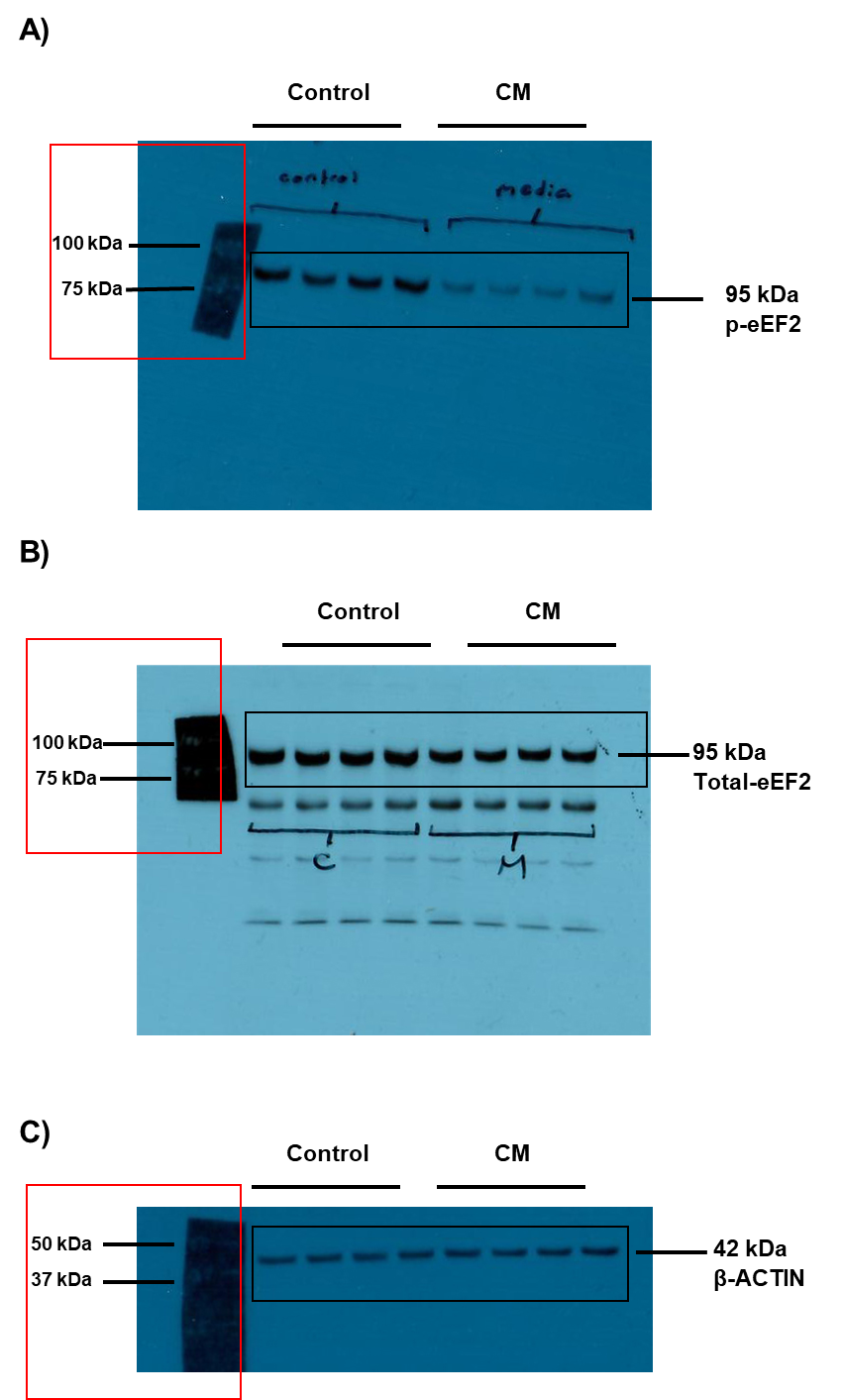


**Supplementary Figure 1. The original, full blot for phosphorylated eukaryotic elongation factor-2 (p-eEF2), total-eEF2 and β-ACTIN.**

**A**: Phosphorylated eukaryotic elongation factor-2 (p-eEF2), **B:** total-eEF2, **C:** β-ACTIN, CM: conditioned medium.
